# Experiences and prospects for cohorts and biobanks

**DOI:** 10.1186/1753-6561-7-S5-O3

**Published:** 2013-08-30

**Authors:** CS Yajnik

**Affiliations:** 1 Diabetes Unit, KEM Hospital, Pune, India

## 

There has been a complete turnaround in scientific thinking about malnutrition as a protective factor to that of a major determinant of diabetes. Development of Type 2 diabetes is attributed to a combination of genetic susceptibility and precipitation factors in adult or early adult life. Current trials and preventive interventions are based on this gene-obesity model causing glucose intolerance necessitating food restriction for management. However when we compare the burden of low birth weight and intrauterine growth restriction with that of type 2 diabetes it can be hypothesised that good nutrition during intrauterine development and early childhood is more important. Foetal programming due to under nutrition can result in intergenerational transfer of long latency disorders.

This hypothesis led to the establishment of the Pune maternal nutrition study and Pune children study involving cohorts. The Pune children study involving 400 children has completed 21 years of follow up and has shown an inverse relationship between birth weight and risk of diabetes mellitus measured by high glucose and high insulin levels. The maternal nutrition study which started with 2675 pregnant women in six villages has now completed 17 years. Both the mothers and children are being followed with very good follow up rates. The study involved consensus building among village leaders, consultation of experts, focus group discussions and training of local girls as Auxiliary Nurse Midwives (ANMs). The study involves assessment of weight and haemoglobin of young women, nutritional intake during pregnancy, foetal growth monitoring, biochemical assessments, storage of maternal and cord blood samples, detailed anthropometry at birth and continuing monitoring for cardiovascular disease risk markers. A steering committee of all stakeholders was constituted to oversee the study. The observations have shown that the intra abdominal fat content of Indian babies is higher. Low vitamin B12 levels, folate levels and high homocysteine levels are shown to be associated with low birth weight and insulin resistance in offspring. This demonstrates that maternal micro nutrient status influences risk of diabetes in the offspring and birth weight is a marker of intrauterine environment. Policy implications include ensuring the health of the young girl and addition of vitamin B12 along with folate for pregnant women. The biochemical estimation was made possible by storage of samples in biobanks which is a most useful resource for subsequent measurements, but there are ethical concerns and issues of subject approval.

